# Supplementary material for: Hierarchical organization of functional connectivity in the mouse brain: a complex network approach
Source: Sci Rep. 2016 Aug 18;6:32060. doi: 10.1038/srep32060 (PMC4989195; doi:10.1038/srep32060)
Supplement: Supplementary Information [file srep32060-s1.pdf]

Hierarchical organization of functional connectivity in the mouse brain: a complex network approach Supplementary Information

Giampiero Bardella[1], Angelo Bifone[2], Andrea Gabrielli[1,3], Alessandro Gozzi[2], and Tiziano Squartini[1,3,*]

[1]Istituto dei Sistemi Complessi ISC-CNR, Universitá "Sapienza" di Roma, P.le A. Moro 5, 00185 Rome, Italy
[2]Italian Institute of Technology, Universitá di Trento, C.so Bettini 31, I-38068 Trento, Italy
[3]IMT Institute for Advanced Studies Lucca, P.zza S. Ponziano 6, 55100 Lucca, Italy
[*]tiziano.squartini@imtlucca.it

ABSTRACT

Supplementary Information of the paper "Hierarchical organization of functional connectivity in the mouse brain: a complex network approach".

ROI - Regions of interest

The list of the neuroanatomical ROI considered for our analysis, together with their abbreviation, is the following (alphabetical order). Fig. 1 shows the ROI mapped into a mouse brain.

1. Acb: accumbens nucleus_dx;
2. Acb: accumbens nucleus_sx;
3. AdHC: anterio-dorsal hippocampus_dx;
4. AdHC: anterio-dorsal hippocampus_sx;
5. Amy: amygdala_dx;
6. Amy: amygdala_sx;
7. Au: auditory cortex_dx;
8. Au: auditory cortex_sx;
9. BF: basal forebrain_dx;
10. BF: basal forebrain_sx;
11. BNST: bed nucleus of stria terminals_dx;
12. BNST: bed nucleus of stria terminals_sx;
13. Cg: cingulate cortex_dx;
14. Cg: cingulate cortex_sx;
15. Collicoli: collicoli_dx;
16. Collicoli: collicoli_sx;
17. Cpu: caudate putamen_dx;
18. Cpu: caudate putamen_sx;

19. DG: dentate gyrus_dx;
20. DG: dentate gyrus_sx;
21. FrA: frontal association cortex_dx;
22. FrA: frontal association cortex_sx;
23. Hypo: hypothalamus_dx;
24. Hypo: hypothalamus_sx;
25. Ins: insular cortex_dx;
26. Ins: insular cortex_sx;
27. M: motor cortex_dx;
28. M: motor cortex_sx;
29. mPFC: medial prefrontal cortex_dx;
30. mPFC: medial prefrontal cortex_sx;
31. OFC: orbitofrontal cortex_dx;
32. OFC: orbitofrontal cortex_sx;
33. Parietal_Ass: parietal association cortex_dx;
34. Parietal_Ass: parietal association cortex_sx;
35. pDG: posterior dentate gyrus_dx;
36. pDG: posterior dentate gyrus_sx;
37. pHC: posterio-ventral hippocampus_dx;
38. pHC: posterio-ventral hippocampus_sx;
39. Pir: piriform cortex_dx;
40. Pir: piriform cortex_sx;
41. Rhinal: rhinal cortex_dx;
42. Rhinal: rhinal cortex_sx;
43. RS: retrosplenial cortex_dx;
44. RS: retrosplenial cortex_sx;
45. S1: primary somatosensory cortex_dx;
46. S1: primary somatosensory cortex_sx;
47. S2: secondary somatosensory cortex_dx;
48. S2: secondary somatosensory cortex_sx;
49. TeA: temporal association cortex_dx;
50. TeA: temporal association cortex_sx;
51. Th: thalamus_dx;
52. Th: thalamus_sx;
53. Vctx: visual cortex_dx;
54. Vctx: visual cortex_sx.

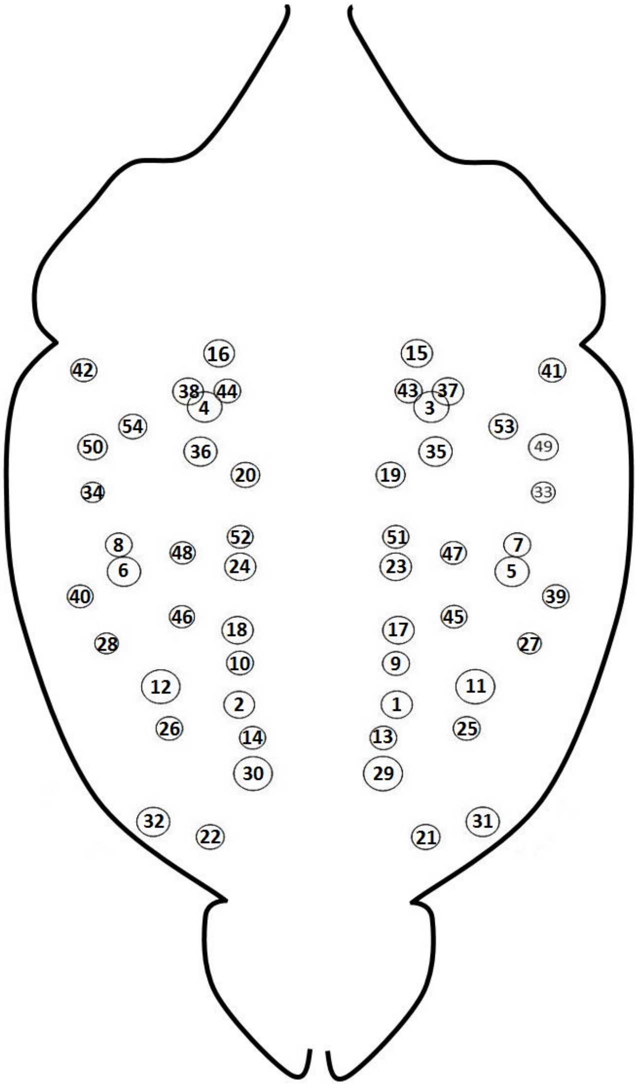

Figure 1. The neuroanatomical ROI considered for our analysis, mapped into a mouse brain..

From time series to correlation matrices

Our data consist of 41 sets of 54 fMRI BOLD-signals each, collected as the time series shown in fig. 2.

The information carried by each mouse-specific set of time series has been condensed into a correlation matrix, whose generic entry C_{ij} is the Pearson coefficient between time series X^i and X^j, defined as

$$\begin{aligned} C_{ij} &= \frac{\mathrm{Cov}[X^i, X^j]}{\sqrt{\mathrm{Var}[X^i] \cdot \mathrm{Var}[X^j]}} = \\ &= \frac{\sum_{t=1}^{T}(X_t^i - m^i)(X_t^j - m^j)}{\sqrt{\sum_{t=1}^{T}(X_t^i - m^i)^2 \cdot \sum_{t=1}^{T}(X_t^j - m^j)^2}} \end{aligned} \quad (1)$$

where $m^i = \frac{\sum_{t=1}^{T} X_t^i}{T}$ and T is the total temporal length of the series.

In order to create an average adjacency matrix describing brain functional connectivity at the population level, subject-wise

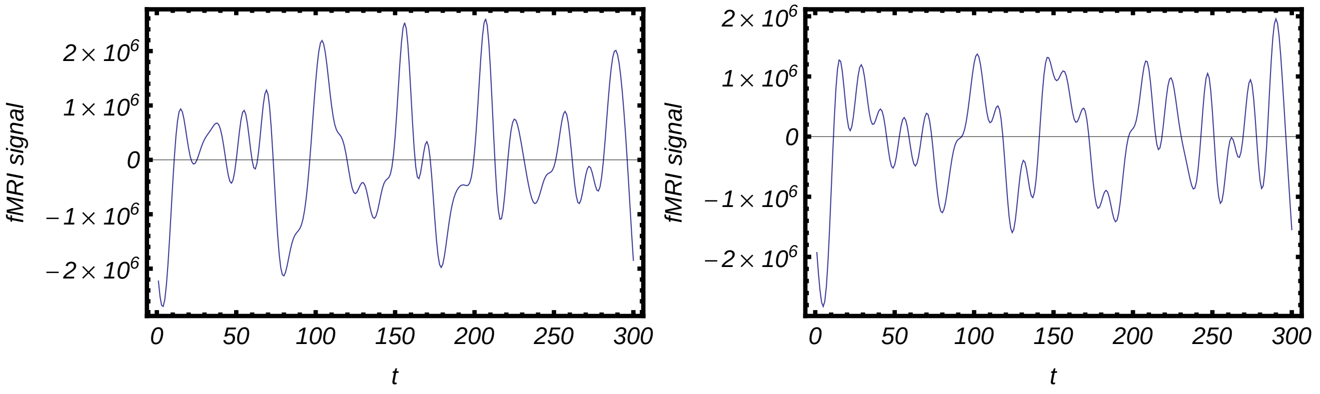

Figure 2. fMRI BOLD-signals corresponding to the right cingulate cortex (top) and left cingulate cortex (bottom) of the brain BE_ag130207a.

matrices were first Fisher-transformed, i.e.

$$z_{ij} = \frac{1}{2}\ln\left(\frac{1+C_{ij}}{1-C_{ij}}\right) = \text{arctanh}(C_{ij}), \tag{2}$$

then averaged across subjects

$$\bar{z}_{ij} = \frac{\sum_{n=1}^{54} z_{ij}^n}{54}, \forall\, i, j \tag{3}$$

(i.e. the generic entry of the average Fisher-transformed matrix is the arithmetic mean of the corresponding individual entries, $z_{ij}^1, z_{ij}^2 \dots z_{ij}^{54}$) and then back-transformed:

$$\overline{C}_{ij} = \tanh(\bar{z}_{ij}), \forall\, i, j. \tag{4}$$

Analysing single individuals

The same analysis described in the main text to study the average correlation matrix has been also applied to the subject-wise matrices, in order to highlight the discrepancies between the individual connectivity structures and the average ones. In what follows, we sum up the results of the analysis of the whole sample of 54 mice, by explicitly showing the plots of four mice only (see figs. **??**-9).

As mentioned in the main test, the empirical cumulative distributions of correlations are always well reproduced by the cumulative density functions of normal distributions whose mean and standard deviation are estimated from the data via a likelihood-maximization procedure.

As for the average mouse brain, our variation of the percolation analysis reveals the presence of plateaus, as well as the existence of a nested structure of highly correlated areas. Analogously, constraining the distribution of correlations allows one to recover the presence of multiple percolation thresholds, even if the brain-specific step-wise plots are not reproduced.

Testing our approach on synthetic networks

In order to test the effectiveness of our method in detecting hierarchical modular structures, we have repeated the same analysis described in the main text to study two synthetic networks.

The first case we considered is a correlation matrix with modular, but not hierarchical, structure. As fig. 7 shows, we considered a network with three well-identifiable modules, weakly inter-connected and lacking an internal hierarchy of correlation coefficients. The dendrogram correctly identifies the presence of three clusters (the different heights are induced by the different values of the internal correlations) with no internal structure; on the other hand, our percolation analysis detects the presence of (three) connected components for lower values of the threshold r (explicitly shown in the bottom panel of fig. 8), while providing an explicit representation of the detachment of single areas in correspondence of higher values. Such a

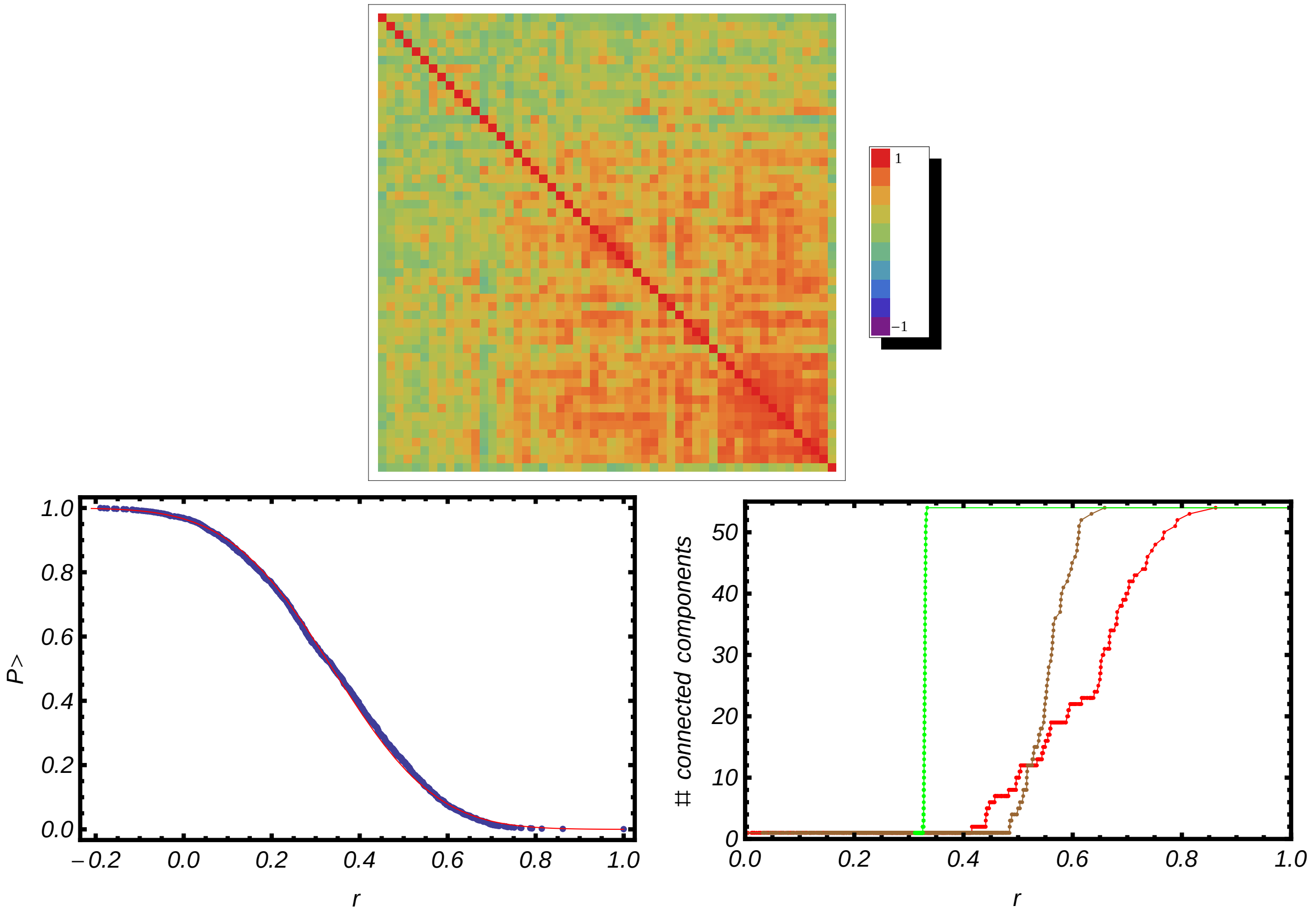

Figure 3. Result of the analysis of an individual brain: (left panels) experimental correlation matrices C_{ij}, whose rows and columns have been ordered according to the dissimilarity measure $D_{ij} = 1 - C_{ij}$, $\forall\, i, j$; (middle panels) empirical cumulative distributions of the correlations - blue trends - and of normal distributions whose parameters have been estimated through the likelihood-maximization procedure - red trends; (right panels) comparison between the result of our modified percolation analysis run on the observed brains - red trends - on the synthetic brains - brown trends - generated by preserving the observed distributions of correlations and averaged on the ensemble - green trends.

behavior signals that a deeper structural organization is, indeed, missing. Notice how such trend differs dramatically from the trend characterizing our average mouse brain, where different regimes are not clearly distinguishable.

The behavior of our second case-study is completely different. In this case, in fact, we have considered a ring of cliques (i.e. not a correlation matrix), each one composed by five nodes, whose internal links are characterized by different values of pseudo-correlation coefficients (see fig. 9). As our percolation analysis reveals, the network progressively breaks down in components characterized by higher internal correlations than with the remaining sub-sets of nodes. As larger correlations are removed, the cliques are correctly recovered as independent modules; further rising the threshold value allows the (internal) modules constituting the cliques to emerge.

References

1. M. P. van den Heuvel, H. E. Hulshoff Pol, *Exploring the brain network: a review on resting-state fMRI functional connectivity*, European Neuropsychopharmacology **20**, 519-534 (2010).
2. Z. Yao, Y. Xie, P. Moore, J. Zheng, *A review of structural and functional brain networks: small world and atlas*, Brain Informatics **2**(9), doi:10.1007/s40708-015-0009-z (2015).
3. E. Bullmore, O. Sporns, *Complex brain networks: graph theoretical analysis of structural and functional systems*, Nature Reviews Neuroscience **10**, 186-198 (2010).
4. O. Sporns, R. Betzel, *Modular brain networks*, Annual Review of Psychology **67**(19), 1-28 (2016).

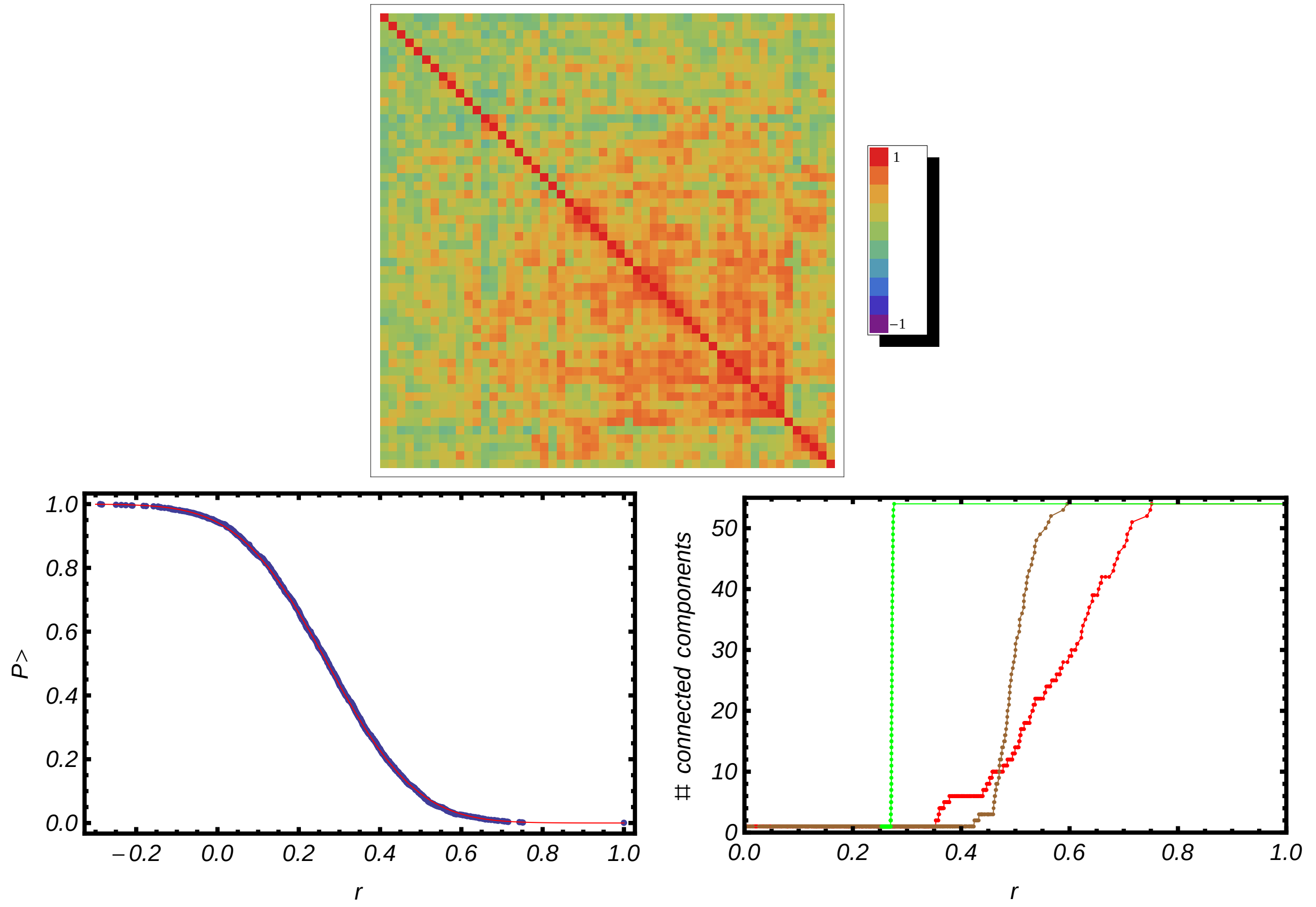

Figure 4. Result of the analysis of an individual brain: (left panels) experimental correlation matrices C_{ij}, whose rows and columns have been ordered according to the dissimilarity measure $D_{ij} = 1 - C_{ij}$, $\forall\, i, j$; (middle panels) empirical cumulative distributions of the correlations - blue trends - and of normal distributions whose parameters have been estimated through the likelihood-maximization procedure - red trends; (right panels) comparison between the result of our modified percolation analysis run on the observed brains - red trends - on the synthetic brains - brown trends - generated by preserving the observed distributions of correlations and averaged on the ensemble - green trends.

5. C. Nicolini, A. Bifone, *Modular structure of brain networks: breaking the resolution limit by surprise*, Scientific Reports **6**(19250), doi:10.1038/srep19250 (2016).

6. P. Moretti, M. Munoz, *Griffiths phases and the stretching of criticality in brain networks*, Nature Communications **4**(2521) (2013).

7. E. Agliari et al., *Retrieval Capabilities of Hierarchical Networks: From Dyson to Hopfield*, Physical Review Letters **114**, 028103 (2015).

8. E. Agliari et al., *Hierarchical neural networks perform both serial and parallel processing*, Neural Networks **66**, 22-35 (2015).

9. E. Agliari et al. *Topological properties of hierarchical networks*, Physical Review E **91**, 062807 (2015).

10. C. Li, H. Wang, W. de Haan, C. J. Stam, P. Van Mieghem, *The correlation of metrics in complex networks with applications in functional brain networks*, Journal of Statistical Mechanics: Theory and Experiment **P11018**, doi:10.1088/1742-5468/2011/11/P11018 (2011).

11. A. Liska, A. Galbusera, A. J. Schwarz, A. Gozzi, *Functional connectivity hubs of mouse brain*, NeuroImage, doi:10.1016/j.neuroimage.2015.04.033 (2015).

12. F. Sforazzini, A.J. Schwarz, A. Galbusera, A. Bifone, A. Gozzi, *Distributed BOLD and CBV-weighted resting-state networks in the mouse brain*, NeuroImage **87**, 403-415 (2014).

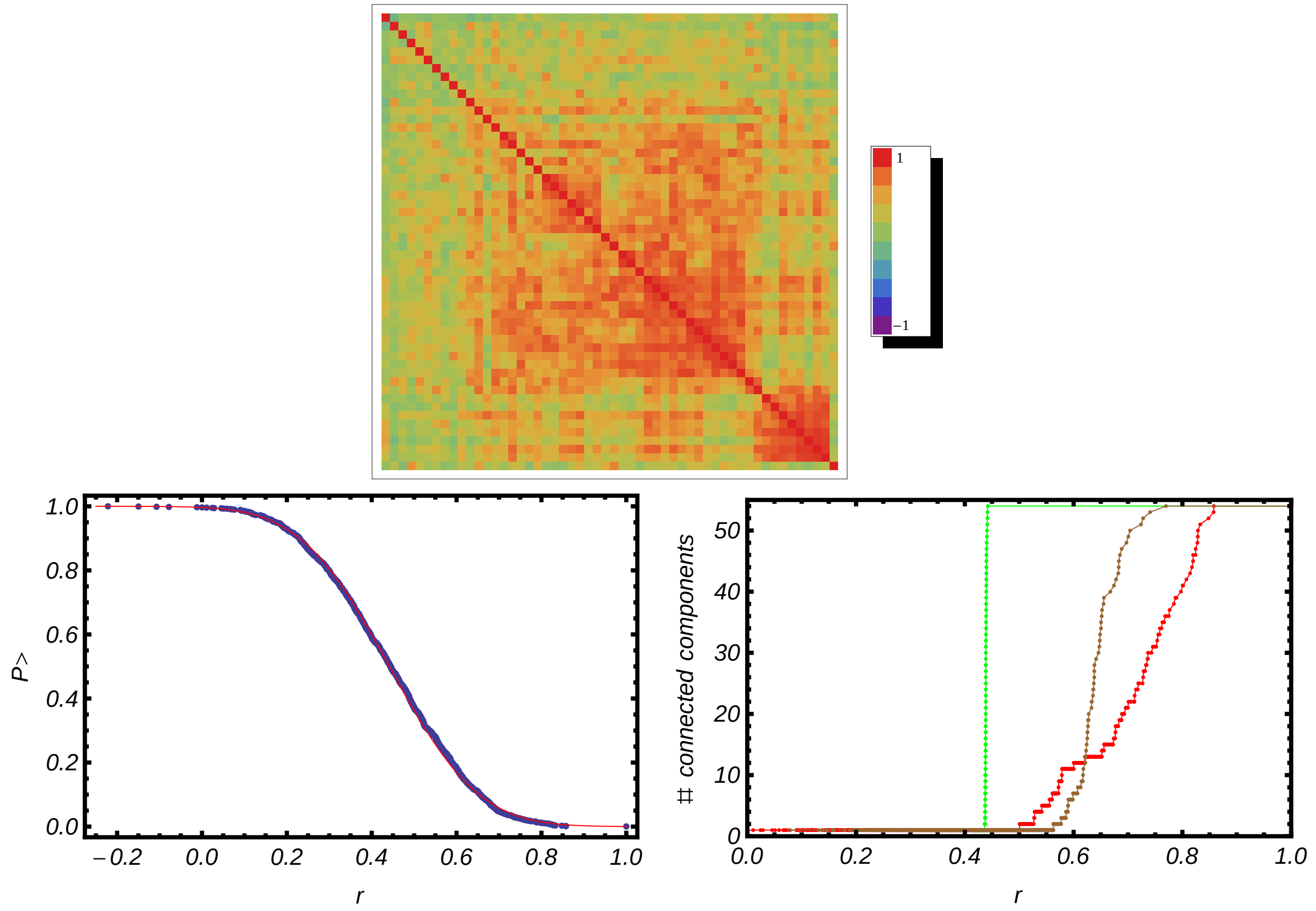

Figure 5. Result of the analysis of an individual brain: (left panels) experimental correlation matrices C_{ij}, whose rows and columns have been ordered according to the dissimilarity measure $D_{ij} = 1 - C_{ij}$, $\forall\, i, j$; (middle panels) empirical cumulative distributions of the correlations - blue trends - and of normal distributions whose parameters have been estimated through the likelihood-maximization procedure - red trends; (right panels) comparison between the result of our modified percolation analysis run on the observed brains - red trends - on the synthetic brains - brown trends - generated by preserving the observed distributions of correlations and averaged on the ensemble - green trends.

13. B. Biswal, F. Z. Yetkin, V. M. Haughton, J. S. Hyde, *Functional connectivity in the motor cortex of resting human brain using echo-planar MRI*, Magnetic Resonance in Medicine **34**, 537-541 (1995).

14. C. Rosazza, L. Minati, *Resting-state brain networks: literature review and clinical applications*, Neurological Science **32**, 773-785, doi:10.1007/s10072-011-0636-y (2011).

15. D. Zhang, M. E. Raichle, *Disease and the brain's dark energy*, Nature Reviews Neurology **6**, 15-28 (2010).

16. M. D. Fox, M. Grecius, *Clinical applications of resting state functional connectivity*, Frontiers in Systems Neuroscience **4**(19) (2010).

17. D. Meunier, R. Lambiotte, E. Bullmore, *Modular and hierarchically modular organization of brain networks*, Frontiers in Neuroscience **4**(200), doi:10.3389/fnins.2010.00200 (2010).

18. S. Achard, R. Salvador, B. Whitcher, J. Suckling, E. Bullmore, *A resilient, low-frequency, small-world human brain functional network with highly connected association cortical hubs*, The Journal of Neuroscience **26**(1), 63-72 (2006).

19. L. K. Gallos, H. A. Makse, M. Sigman, *A small world of weak ties provides optimal global integration of self-similar modules in functional brain networks*, Proceedings of the National Academy of Science **109**(8), 2825-2830 (2012).

20. A. Bifone, A. Gozzi, A. J. Schwarz, *Functional connectivity in the rat brain: a complex network approach*, Magnetic Resonance Imaging, 1200-9, doi:10.1016/j.mri.2010.07.001 (2010).

21. H. Simon, *The architecture of complexity*, Proceedings of the American Philosophical Society **106**(6), 467-482 (1962).

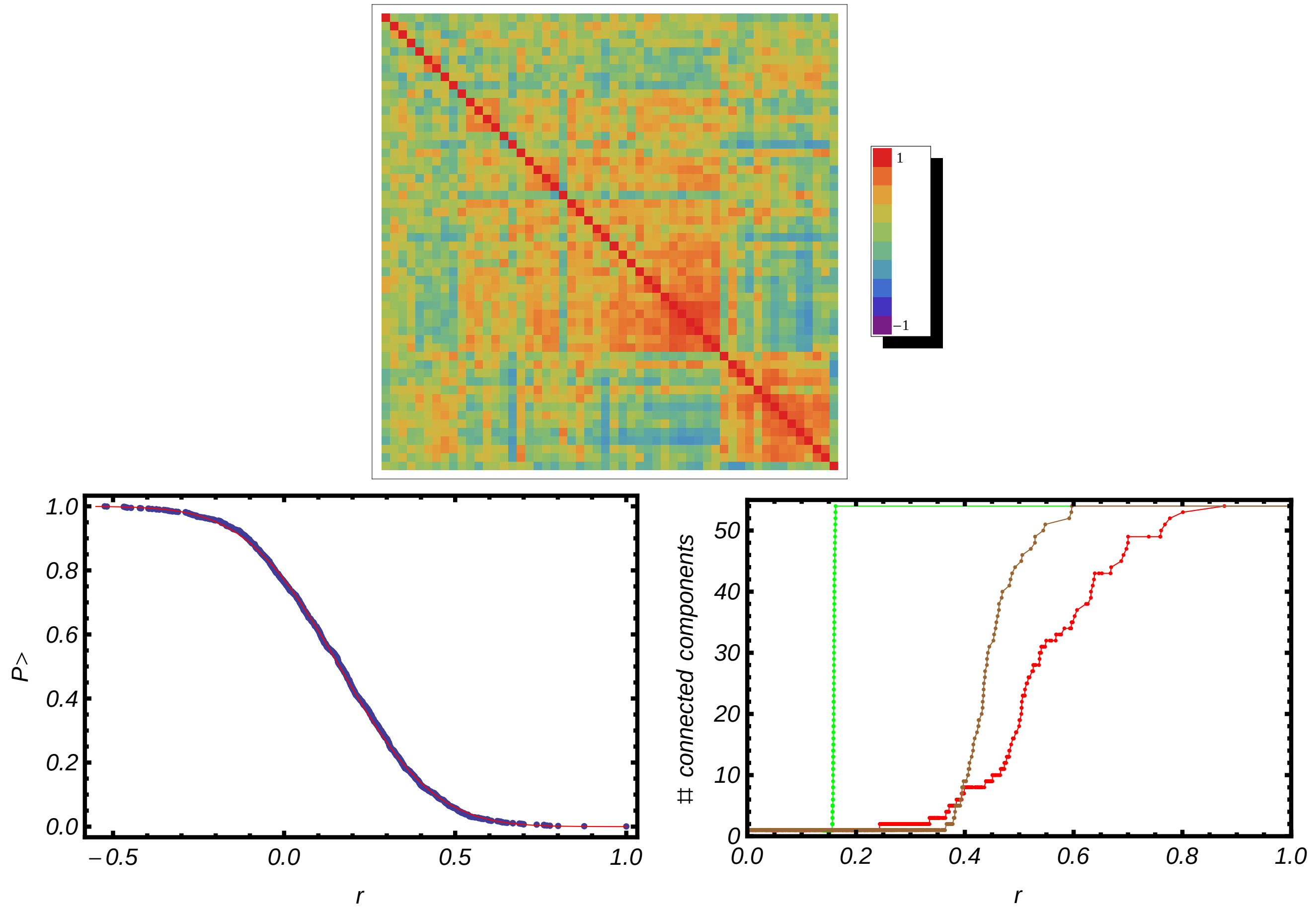

Figure 6. Result of the analysis of an individual brain: (left panels) experimental correlation matrices C_{ij}, whose rows and columns have been ordered according to the dissimilarity measure $D_{ij} = 1 - C_{ij}$, $\forall\, i, j$; (middle panels) empirical cumulative distributions of the correlations - blue trends - and of normal distributions whose parameters have been estimated through the likelihood-maximization procedure - red trends; (right panels) comparison between the result of our modified percolation analysis run on the observed brains - red trends - on the synthetic brains - brown trends - generated by preserving the observed distributions of correlations and averaged on the ensemble - green trends.

22. N. Chatterjee, S. Sinha, *Understanding the mind of a worm: hierarchical network structure underlying nervous system function in C. elegans*, in Progress in Brain Research **168**(12), 145-153 (2008).

23. J. G. White, E. Southgate, J. N. Thomson, S. Brenner, *The structure of the nervous system of the nematode C. elegans*, Philosophical Transactions of the Royal Society of London B **314**, 1-340 (1986).

24. R. Albert, A.-L. Barabasi, *Statistical mechanics of complex networks*, Reviews of Modern Physics **74**, 47-97 (2002).

25. B. Bollobas, *Random Graphs*, Cambridge University Press (2001).

26. M. E. J. Newman *Finding community structure in networks using the eigenvectors of matrices*, Physical Review E **69**, 026113 (2004).

27. M. E. J. Newman, M. Girvan *Finding and evaluating community structure in networks*, Physical Review E **69**, 026113 (2004).

28. M. MacMahon, D. Garlaschelli *Community detection for correlation matrices*, Physical Review X **5**(021006) (2015).

29. S. M. Hadi Hosseini, S. R. Kesler, *Influence of choice of null network on small-world parameters of structural correlation networks*, PLoS ONE **8**(6): e67354 (2013).

30. http://fsl.fmrib.ox.ac.uk/fsl/.

31. M. Jenkinson, C. F. Beckmann, T. E. Behrens, M. W. Woolrich, S. M. Smith, *Fsl*, NeuroImage **62**, 782-790 (2012).

32. http://afni.nimh.nih.gov/afni/.

33. F. Sforazzini et al. *Altered functional connectivity networks in acallosal and socially impaired BTBR mice*, Brain Structure and Function, 1-14 (2014).

34. N. J. Higham, *Computing the nearest correlation matrix - a problem from finance*, IMA Journal of Numerical Analysis **22**, 329-343 (2002).

35. F. Murtagh, P. Contreras, *Algorithms for hierarchical clustering: an overview*, Wiley Interdisciplinary Reviews: Data Mining and Knowledge Discovery **2**(1), 86-97 (2012).

36. S. D. Vann, J. P. Aggleton, E. A. Maguire, *What does the retrosplenial cortex do?*, Nature Reviews **10**, 792-803 (2009).

37. D. Choi et al. *Bed nucleus of the stria terminalis subregions differentially regulate hypothalamic-pituitary-adrenal axis activity: implications for the integration of limbic inputs*, Journal of Neuroscience **27**(8) (2007).

38. L. R. Squire, C. E. Stark, R. E. Clark, *The medial temporal lobe*, Annual Review of Neuroscience **27**, 279-306 (2004).

39. M. G. Kitzbichler, M. L. Smith, S. R. Christensen, E. Bullmore, *Broadband criticality of human brain network synchronization*, PLoS Computational Biology **5**: e1000314 (2009).

40. P. A. Robinson, J. A. Henderson, E. Matar, P. Riley, R. T. Gray, *Dynamical reconnection and stability constraints on cortical network architecture*, Physical Review Letters **103** 108104, (2009).

41. E. Bullmore, O. Sporns, *The economy of brain network organization*, Nature Reviews. Neuroscience, **13**(5), 336–49, doi:10.1038/nrn3214 (2012).

42. A. F. Alexander-Bloch et al. *Disrupted modularity and local connectivity of brain functional networks in childhood-onset schizophrenia*. Frontiers in System Neuroscience **4**(147), doi:10.3389/fnsys.2010.00147 (2010).

Acknowledgements

A.G. and T.S. acknowledge support from the EU project FET-Open FOC (grant num. 255987), the FET project SIMPOL (grant num. 610704) and the FET project DOLFINS (grant num. 640772). A.B. acknowledges funding from the European Union's Horizon 2020 research and innovation programme under grant agreement num. 668863.

Author contributions statement

A.B., A.G., A.G. and T.S. conceived the analysis. G.B. conducted the analysis. All authors reviewed the analysis. A.B. and T.S. wrote the paper. All authors reviewed the manuscript.

Additional information

Competing financial interests. The authors declare no competing financial interests.

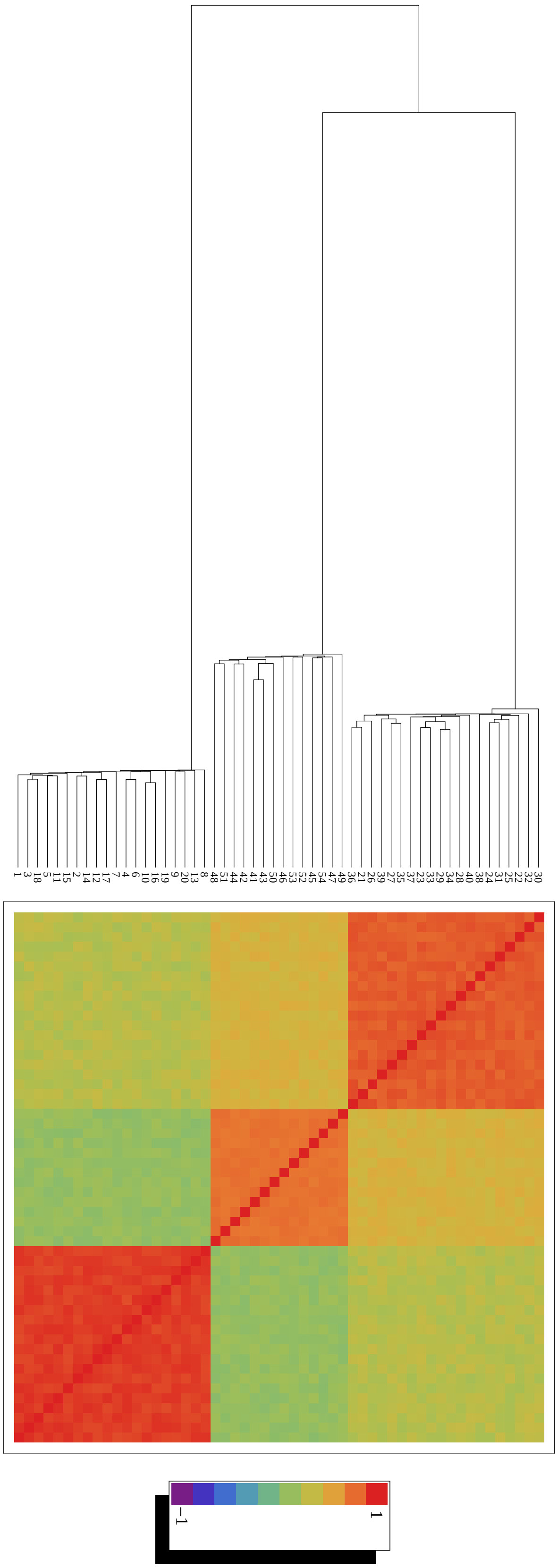

Figure 7. Dendrogram and correlation matrix of a synthetic correlation matrix with three well-identifiable clusters. Notice that clusters are weakly-interconnected and lack a hierarchical (internal) structure.

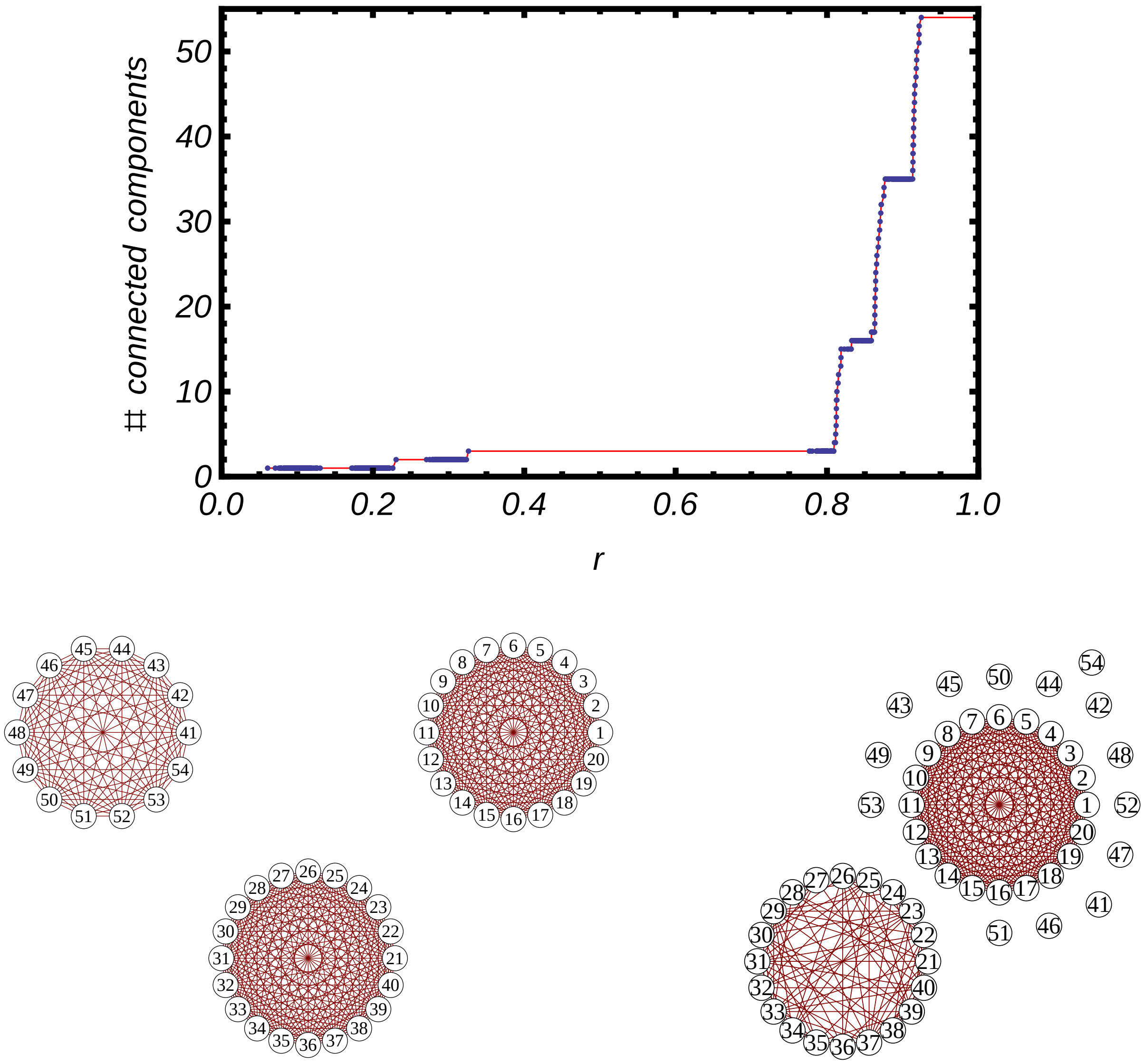

Figure 8. Result of the percolation analysis on our synthetic correlation matrix with three well-identifiable, non-hierarchical, clusters. Our modified percolation detects the presence of (three) connected components for lower values of the threshold r, while providing an explicit representation of the detachment of single areas in correspondence of higher values. Bottom panel shows the modules recovered in correspondence of $r \simeq 0.35$ and the detachment of single areas for $r \simeq 0.85$.

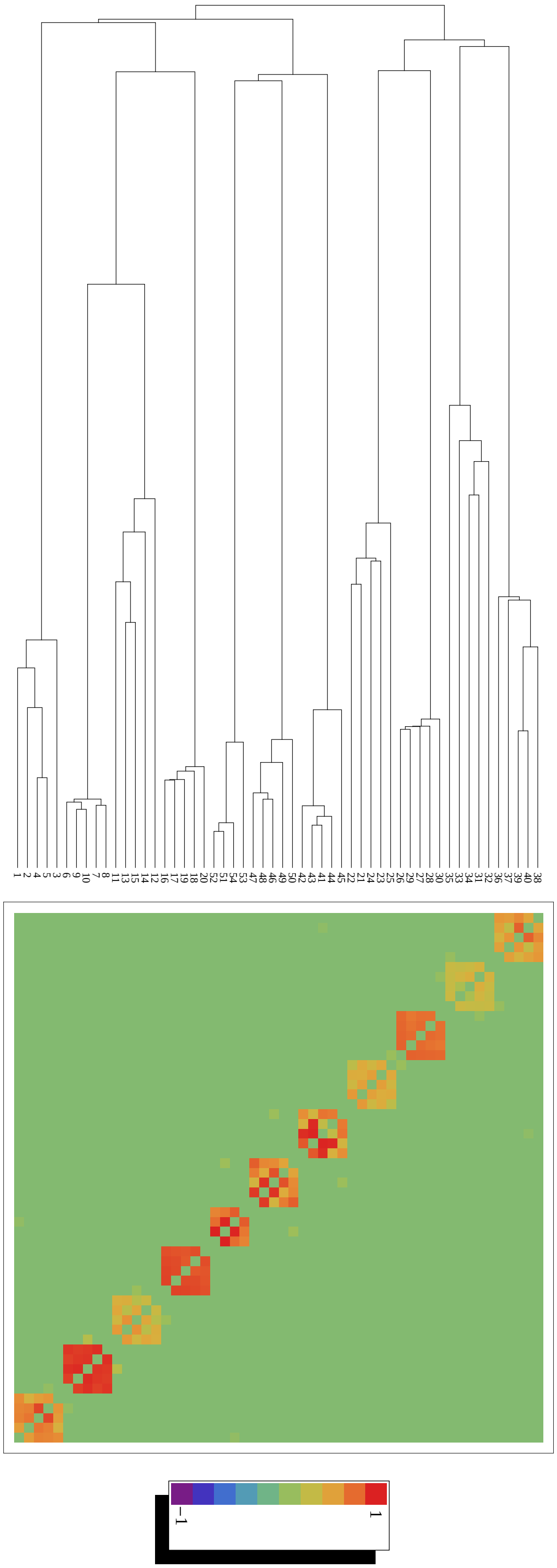

Figure 9. Dendrogram and adjacency matrix of a synthetic network characterized by a ring of cliques structure, whose nodes are linked by pseudo-correlation coefficients, ranging between 0 and 1.

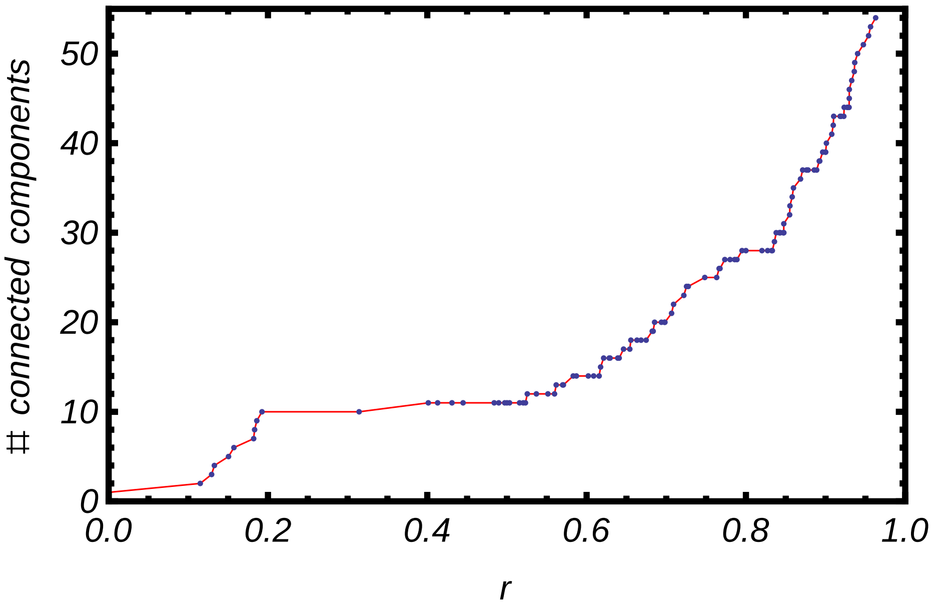

Figure 10. Result of the percolation analysis on our synthetic network characterized by a ring of cliques structure. Our modified percolation detects the presence of modules within modules, characterized by higher internal correlations than with the remaining sub-sets of nodes.

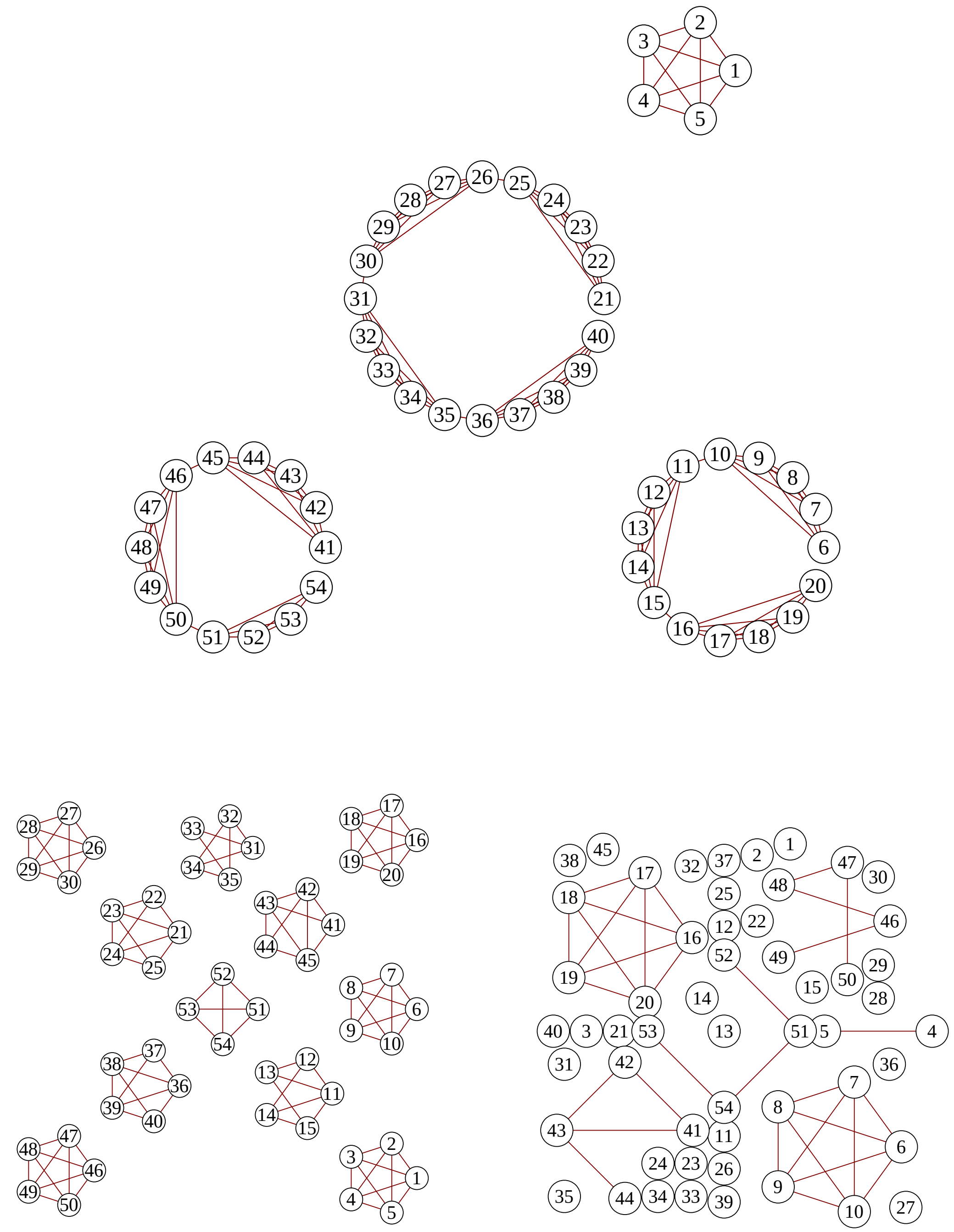

Figure 11. Explicit representation of the modules recovered by our percolation analysis on the ring of cliques. Our modified percolation detects the presence of modules within modules (for $r \simeq 0.15$), characterized by higher internal correlations than with the remaining sub-sets of nodes. As larger correlations are removed, the single cliques are correctly recovered ($r \simeq 0.5$); further rising the threshold value ($r \simeq 0.86$) allows the internal modules constituting the cliques to emerge.
